# Supplementary material for: Sustained-input switches for transcription factors and microRNAs are central building blocks of eukaryotic gene circuits
Source: Genome Biol. 2013 Aug 23;14(8):R85. doi: 10.1186/gb-2013-14-8-r85 (PMC4054853; doi:10.1186/gb-2013-14-8-r85)
Supplement: Additional file 5 — HTML Browsable Motif Output. Zipped folder containing all WaRSwap and FANMOD motif output, viewable in a web browser. [file gb-2013-14-8-r85-S5.ZIP › HTML_browsable_motif_output/WaRSwap_ath_tair9/sigs_WSWRSLDraw_2-2000.pvals.heatmaps.html/motif_id_36_001011002_tftype_ath_upstream_-1000_0.html]

```
BG_MODEL = Rnew2
MOTIF_ID = 36_001011002
TF_TYPE = ath
UPSTREAM = -1000_0


PVals
FN_0.2	FN_0.4	FN_0.6	FN_0.8
dg_60.genes	0.007	0.053	0.992	1
dg_70.genes	0.01	0.079	0.986	1
dg_80.genes	0.009	0.107	0.984	1

ZScores
FN_0.2	FN_0.4	FN_0.6	FN_0.8
dg_60.genes	2.74	1.713	-2.664	-10.431
dg_70.genes	2.537	1.558	-2.52	-10.387
dg_80.genes	2.955	1.396	-2.15	-7.937

StDevs
FN_0.2	FN_0.4	FN_0.6	FN_0.8
dg_60.genes	2858.365	1373.918	546.437	322.131
dg_70.genes	2188.02	1013.651	414.313	236.502
dg_80.genes	874.929	449.95	224.698	158.359
```
